# Long-term trends in co-occurring medical and psychiatric hospitalizations among children and adolescents in Ontario, Canada.

**DOI:** 10.23889/ijpds.v9i5.2525

**Published:** 2024-09-18

**Authors:** Natasha Saunders, Astrid Guttmann, Maria Chiu, Sima Gandhi, Simone Vigod, Paul Kurdyak, Kinwah Fung, Isobel Sharpe, Scott Emerson, Alene Toulany

**Affiliations:** 1 The Hospital for Sick Children; 2 ICES; 3 University of Toronto; 4 Women's College Hospital; 5 Centre for Addiction and Mental Health

## Background

Psychiatric conditions are common amongst hospitalized children. Co-occurring psychiatric conditions for medical hospitalizations contribute to length of stay, costs, and readmissions. We sought to measure trends over 20 years in pediatric hospitalizations for co-occurring medical and psychiatric conditions and compare with those without psychiatric comorbidity, overall and in free-standing children’s hospitals.

## Methods

We identified all 3- to 17-year-olds hospitalized in Ontario, Canada between April 1, 2003 and March 31, 2022. Using health record discharge diagnoses, hospitalizations were assigned to 1 of 4 groups: 1) medical-diagnosis-only, 2) psychiatric-diagnosis-only, 3) primary medical diagnosis with psychiatric comorbidity, and 4) primary psychiatric diagnosis with medical comorbidity. Hospitalization trends for 1) all hospitals, and 2) free-standing children’s hospitals were described and compared.

## Results

From 2003 to 2022, medical-diagnosis-only hospitalizations declined 39% (41,909 to 25,486 hospitalizations), psychiatric-diagnosis-only hospitalizations increased 96% (3227 to 6337), medical hospitalizations with psychiatric comorbidity increased 127% (977 to 2221) and psychiatric hospitalizations with medical comorbidity increased 100% (2051 to 4096). Among pediatric hospitals, medical-diagnosis-only hospitalizations increased 23% (12,430 to 15,318), psychiatric-diagnosis-only hospitalizations increased 420% (271 to 1408), psychiatric hospitalizations with medical comorbidity increased 172% (539 to 1468) and medical hospitalizations with psychiatric comorbidity increased 235% (478 to 1599).

## Conclusions

Hospitals have experienced large absolute and relative increases in volumes for psychiatric conditions both with and without co-occurring medical conditions, particularly among free-standing children’s hospitals. Healthcare provider training, hospital resourcing, and health system planning must consider how best to accommodate the increasing acute psychiatric care needs of hospitalized children and adolescents.

